# Amygdala Corticofugal Input Shapes Mitral Cell Responses in the Accessory Olfactory Bulb

**DOI:** 10.1523/ENEURO.0175-18.2018

**Published:** 2018-06-11

**Authors:** Livio Oboti, Eleonora Russo, Tuyen Tran, Daniel Durstewitz, Joshua G. Corbin

**Affiliations:** 1 Center for Neuroscience Research, Children's National Health System, Washington, DC 20010; 2 Department of Theoretical Neuroscience, Bernstein Center for Computational Neuroscience, Central Institute of Mental Health, Medical Faculty Mannheim of Heidelberg University, 68159 Mannheim, Germany

**Keywords:** accessory olfactory bulb, amygdala, circuitry, connectivity, mitral cells

## Abstract

Interconnections between the olfactory bulb and the amygdala are a major pathway for triggering strong behavioral responses to a variety of odorants. However, while this broad mapping has been established, the patterns of amygdala feedback connectivity and the influence on olfactory circuitry remain unknown. Here, using a combination of neuronal tracing approaches, we dissect the connectivity of a cortical amygdala [posteromedial cortical nucleus (PmCo)] feedback circuit innervating the mouse accessory olfactory bulb. Optogenetic activation of PmCo feedback mainly results in feedforward mitral cell (MC) inhibition through direct excitation of GABAergic granule cells. In addition, LED-driven activity of corticofugal afferents increases the gain of MC responses to olfactory nerve stimulation. Thus, through corticofugal pathways, the PmCo likely regulates primary olfactory and social odor processing.

## Significance Statement

Olfactory inputs are relayed directly through the amygdala to hypothalamic and limbic system nuclei, regulating essential responses in the context of social behavior. However, it is not clear whether and how amygdala circuits participate in the earlier steps of olfactory processing at the level of the olfactory bulb. Unraveling the organization of this circuitry is critical to understand the function of amygdala circuits. Combining *cre*-dependent viral tracing with optogenetic-assisted patch-clamp electrophysiology, the present work maps the synaptic connectivity and physiology of a cortical amygdala pathway innervating primary olfactory circuits.

## Introduction

The accessory olfactory system (AOS) plays a crucial role in the detection of sensory signals used for individual recognition in the context of social, reproductive and parental relationships (Winans and Powers, 1977; Halpern, 1987; Meredith, 1991; Dulac and Wagner, 2006). Accessory olfactory bulb (AOB) neurons processing these chemical signals relay their output directly to the amygdala, which in turn provides feedback projections to AOB circuits (Raisman, 1972). Although the precise cell-to-cell connectivity of these circuits is largely unknown, the lack of thalamic relays implies that any refinement of the incoming sensory information must be conducted by primary AOS circuits, amygdala feedback projections, or both.

The AOS detects olfactory information through sensory neurons localized in the vomeronasal organ (VNO). Each sensory neuron innervates multiple glomeruli in the AOB, the most posterior-dorsal bulbar region (Belluscio et al., 1999). Here, mitral cells (MCs) integrate inputs from multiple glomeruli (Wagner et al., 2006) before relaying this information directly to the medial amygdala (MeA) and cortical [posteromedial cortical nucleus (PmCo)] amygdala subnuclei (Winans and Scalia, 1970). Importantly, this connectivity differs dramatically from the main olfactory bulb (MOB), where each MC contacts a single glomerulus composed of input from sensory neurons expressing the same receptor subclass. Therefore, whereas in the MOB each MC primarily encodes inputs from single odorants, AOB MCs convey to the amygdala related blends of chemical ligands, which can be as complex as the number of afferent receptor neurons on a given MC. Surprisingly, AOB MCs are capable of highly selective responses to complex individual odor signatures (Luo et al., 2003; Ben-Shaul et al., 2010), yet how such narrow tuning is achieved is unclear. Among the possible mechanisms, lateral inhibition through local GABAergic granule interneurons [granule cells (GCs)] has been proposed for both the MOB and AOB (Hendrickson et al., 2008; Geramita et al., 2016). In the MOB, in addition to these horizontal interactions, GC activity is also strongly modulated by top-down feedback from the piriform cortex (Balu et al., 2007; Matsutani, 2010; Boyd et al., 2012). Not only has it become increasingly evident that this modulatory feedback represents a critical component of olfactory perception (Boyd et al., 2012; Markopoulos et al., 2012; Otazu et al., 2015; Oettl et al., 2016), but it is also clear that both mechanisms can interact to generate optimized odor representations by MCs.

Here, we dissect the functional connectivity of a corticobulbar amygdala circuit originating in the PmCo and modulating (AOB) output neurons. We show that PmCo input indirectly modulates MC firing through local inhibitory networks. This occurs via enhancement of MC responses to electrically evoked vomeronasal inputs from the periphery. Our results reveal that modulatory feedback from the cortical amygdala is capable of exerting top-down modulation likely on peripheral AOS responses to social stimuli.

## Materials and Methods

### Animals

Mice were housed in the Children’s National Health Center temperature- and light-controlled animal care facility and given food and water *ad libitum*. All animal procedures were approved by the Children’s National Institutional Animal Care and Utilization Committee and conformed to National Institutes of Health Guidelines for animal use. *nNOS^cre^* mice (B6.129-Nos1tm1(cre)Mgmj/J; RRID:SCR_014588), *RABV* mice (B6;129P2-Gt(ROSA)26Sortm1 (CAG-RABVgp4,-TVA)Arenk/J; stock #024708), *GAD^cre^* mice (Gad2 < tm2(cre)Zjh>/J; RRID:MGI:4418723), and *Dlx5/6^cre^* mice (Tg(dlx6a-cre)1Mekk/J; RRID:IMSR_JAX:008199) were all obtained from The Jackson Laboratory. *Sim1^cre^* mice were provided by Joel Elmquist (Tg(Sim1-cre)1Lowl/J; RRID:IMSR_JAX:006395), and *Pcdh21^cre^* animals were provided by Dr. Kevin Briggman [Tg(Cdhr1-cre) KG76Gsat; RRID:MMRRC_036074-UCD].

### Viral vectors and stereotaxic injections

The following procedures were followed for each tracer or viral vector injected: postpubertal mice (postnatal day 30–50) were anesthetized with an intraperitoneal injection of a 10 μl/g of anesthetic cocktail (8.5 ml sterile saline, 1 ml 100 mg/ml ketamine, 0.5 ml 20 mg/ml xylazine). Injection sites targeting the PmCo were determined based on coordinates that referred to bregma: X, −2.5; Y, 2.6; Z, −5.3. Injections (50–100 nl) were made bilaterally using beveled glass pipettes (Kingston Glass) at depths of 5.1–5.3 mm from the pial surface. Viral injections were manually assisted by the use of a Pico Injector (catalog #pli-100, Harvard Apparatus), each pressure step delivering 10–20 nl, 1 per minute. Ten minutes after the final injection, the glass pipette was withdrawn and the wound sutured. Pseudotyped rabies virus (PRV) tracing from the AOB was preferably performed using the *RABV* mouse line due to problems encountered with tissue damage and starter cell viability, especially in AOB GCs.

Cholera toxin subunit-B (Ct-b; Alexa Fluor 555 Conjugate, C34776; Alexa Fluor 488 Conjugate, C22841; Thermo Fisher Scientific) was diluted 10 μg/μl in sterile PBS, aliquoted, and stored at 4°C until use. The following viral vectors were obtained as follows: University of North Carolina Vector Core: double-floxed reporter, rAAV5/EF1a-DIO-eYFP; University of Pennsylvania Vector Core: double-floxed channelrhodopsin 2 (ChR2), AAV9.EF1.dflox.hChR2 (H134R)-mCherry.WPRE.hGH, AddGene20297; CaMKIIa-ChR2, AAV1.CaMKIIa.hChR2 (H134R)-mCherry.WPRE.hGH; Salk Institute Vector Core: G-deleted rabies, PRV, AddGene 32635 (eGFP), 32636 (mCherry). Each vector was aliquoted and stored at −80°C until use.

### Histology and immunohistochemistry

Mice were anesthetized with a 4:1 cocktail of ketamine and xylazine (Bayer) and perfused transcardially with 0.9% saline solution followed by 4% paraformaldehyde in 0.1 m PBS. Brains were removed, postfixed for 6 h in 4% paraformaldehyde, and incubated overnight in 0.1 m PBS containing 30% sucrose. Cryosections (30 μm thick) were mounted on SuperFrost Plus glass slides for immunofluorescence analysis. Tissue sections were washed (10 min) in PBS; incubated in blocking solution containing 0.5% Triton X-100, 4% horse serum, and PBS (1 h, room temperature); and incubated overnight at 4°C in blocking solution containing the first primary antibody. Tissue was then washed in PBS (10 min), followed by incubation in secondary antibody for 1 h at room temperature. The primary antibodies used were as follows: anti-Tbr1 (1:500, chicken polyclonal; catalog #AB2261, Millipore); anti-CaMKIIa (1:500; mouse; catalog #SA-162, Biomol Research Laboratories); anti-Sim1 (1:1000; rabbit; catalog #ab4144, Millipore; RRID:AB_2187608); anti-Cux1 (1:100; mouse; catalog #sc-514008, Santa Cruz Biotechnology). The secondary antibodies used were as follows: Alexa Fluor 488 donkey anti-mouse (RRID:AB_141607); Alexa Fluor 647 donkey anti-chicken (RRID:AB_11194678); and Alexa Fluor 647 donkey anti-rabbit (RRID:AB_2536183; all diluted 1:1000).

### Brain 3D reconstructions

**Figure 1.**
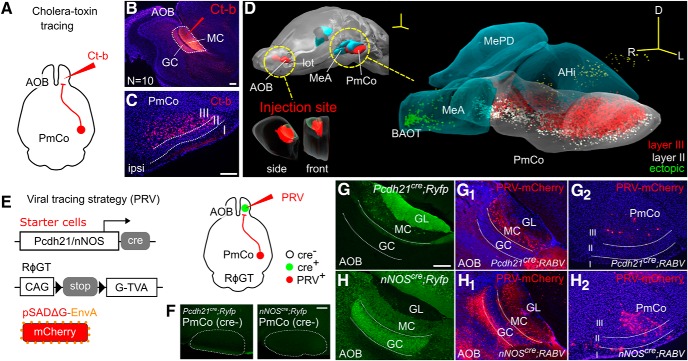
ACPs to the AOB arise in the PmCo. ***A–C***, Ct-b injections (***A***) targeting the AOB (***B***) result in retrograde labeling in the PmCo (***C***). ***D***, 3D serial section reconstruction of the medial and cortical amygdala showing the extent of a typical Ct-b injection in the AOB. ***E***, PRV retrograde viral-tracing strategy used to identify the putative synaptic targets of PmCo projection neurons in the AOB. Cre-expressing “starter cells” are defined as those activating PRV retrograde infection in the AOB. ***F***, As Cre expression is limited to AOB starter cells and is absent in the PmCo (***F***), PRV spreads retrogradely across one synapse only. ***G***, ***H***, Starter neurons are either MCs (in *Pcdh21^cre^;RABV* mice (***G***) or GABAergic nNOS-expressing cells in the GC layer (GC; in *nNOS^cre^;RABV* mice; ***H***). PRV injections in the AOB of *Pcdh21^cre^;RABV* or *nNOS^cre^;RABV*cre mice results in local infection (***G_1_***, ***H_1_***) and monosynaptic retrograde spread to the PmCo (***G_2_***, ***H_2_***). Scale bars: ***A***, ***B***, ***G–H_2_***, 200 μm; ***C***, 50 μm; ***D***, 500 μm). Abbreviations: MeA, medial amygdala; MePD, medial amygdala posterodorsal subdivision; BAOT, bed nucleus of the accessory olfactory tract; AHi, amygdala hippocampal transition area; GL, glomerular layer.

The 3D reconstructions of injected brains or areas (Fig. 1, spatial representation of Ct-b staining; also see Fig. 5, viral expression) were obtained by assembling stacks of images acquired from seriate and consecutive brain sections (30 µm thick) using the ImageJ “TrackEM2” plugin. The 3D morphology of Ct-b or viral labeling was captured by 2D thresholded contour delineation. The import of the 3D assembly into the open source software Blender (https://www.blender.org/) allowed the editing of shading, transparency, lighting, and 3D rendering of the reconstruction.

### Acute brain slice preparation

Acute slices were prepared from 2- to 4-month-old male and female mice. Animals were anesthetized with CO_2_ and decapitated. Brains were removed quickly and placed in cold (48°C) sucrose-based oxygenated (95% O_2_/5% CO_2_) cutting solution composed of the following (in mm): sucrose 234, glucose 11, NaHCO3 26, KCl 2.5, NaH_2_PO_4_ H2O 1.25, MgSO4 7 × H2O 10, and CaCl2 H_2_O 0.5. Coronal slices containing the PmCo were obtained with a slicing vibratome (VT1200s, Leica) by removing the cerebellum with a perpendicular cut to the rostral–caudal plane and gluing the caudal side down on the vibratome stage submerged in cold cutting solution. The slice thickness was 300 um for all experiments. The slices were immersed in oxygenated (95% O_2_/5% CO_2_) artificial CSF (ACSF) at 34°C for 30–45 min. ACSF was composed of the following (in mm): NaCl 126, NaHCO3 26, glucose 10, KCl 2.5, NaH_2_PO_4_ H2O 1.25, MgCl2 7 × H_2_O 2, and CaCl2 2 × H2O 2, pH 7.4, with osmolarity maintained at 290–300 mOsm.

### Slice electrophysiology

Slices were transferred to a recording chamber and superfused with ACSF. All experiments were conducted at room temperature 25–27°C. Patch-clamp recordings were performed using an upright microscope (model E600 F, Nikon), equipped with 10× and 60× objectives and differential interference contrast optics. Neuron types were identified by their morphology, intrinsic properties, and layering within the different nuclei examined (OB or PmCo). In some recordings, biocytin (3–5%; B1592, Thermo Fisher Scientific) was added to the intracellular solution. This contained the following (in mm): 130 K-gluconate, 10 NaCl, 10 HEPES, 0.6 EGTA, 2 Na_2_ATP, and 0.3 Na_3_GTP. In some cases, when inhibitory currents were recorded (pair recording experiments) the following high-chloride solution was used: 70 K-gluconate, 70 KCl, 10 HEPES, 10 EGTA, 2 MgCl_2_, 2 Na_2_ATP, and 0.3 Na_3_GTP. Recordings were made using a Multiclamp 700B Amplifier (Molecular Devices) digitized at 10–20 kHz and acquired using Clampex Software (pClamp 10, Molecular Devices). For most recordings, pipette resistance was 3–6 MΩ. Series resistance was normally <30 MΩ and periodically monitored. Bessel was set at 1 kHz for all voltage-clamp and 10 kHz for current-clamp experiments. Gain was set at 5 V/V in current-clamp recordings. For experiments involving optogenetic stimulations, a patterned LED light illuminator (Polygon 400, Mightex) was used to illuminate tissue sections (light source, 470 nm, 11 mW; Mightex). Full-field illumination was used unless stated otherwise, setting the LED intensity at 10% of the maximum, which gave us the best control on LED spatial specificity. During MC recordings, GC stimulation was obtained by centering the objective on the GC layer, just below the recorded MCs but far enough to avoid mitral layer stimulations. Full-field illumination did not alter the amplitude of light-evoked responses. The stimulation frequencies used during paired recordings were chosen to mimic odor-evoked responses (Schoppa, 2006) and, in the case of optogenetic activation, to elicit efficient ChR2-mediated AP propagation while avoiding channel habituation (Lin, 2011).

### Protocol used for dual vomeronasal nerve and PmCo stimulations

**Figure 2.**
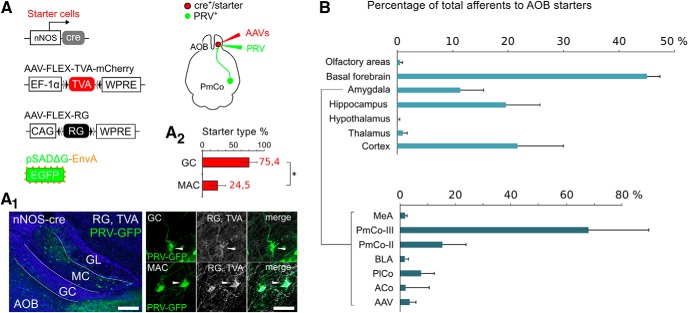
ACPs are synaptically connected to cells in the AOB GC layer. ***A***, Conditional PRV tracing with helper adeno-associated viruses used to identify starter cells in the AOB GC layer. ***A_1_***, Both GCs and macs are identified as starters (TVA-G/PRV^+^). ***A_2_***, Estimate percentage contribution of each cell type (macs or GCs) to the total amount of starter cells in the GC layer (paired *t* test, *p* < 0.05). ***B***, The top histograms (light gray) show the percentage of input neurons (PRV labeled) to the AOB GC layer per brain area, referred to the total amount of PRV-labeled neurons in the brain (*N* = 4 brains). The histogram below (dark gray) show the percentage of input neurons relative to the amount of PRV-labeled neurons within the amygdala only. Layer II and III of the PmCo provide the highest amount of inputs. Scale bars: ***A_1_***, left, 200 μm; ***A_1_***, right, 20 μm. GL, glomerular layer; BLA, basolateral amygdala; PlCo, Posterolateral cortical amygdala; ACo, anterior cortical amygdala; aav, anterior amygdala, ventral subdivision.

Mitral cells were recorded during the following four different conditions: (1) spontaneous activity was recorded in absence of any stimulation (“baseline”); (2) mitral cell firing was recorded in presence of glomerular electrode stimulation only (“E”), using a stimulation frequency previously used to mimic the physiologic activity of olfactory afferents (100 Hz trains at 4 Hz; Schoppa, 2006); and (3) MCs were recorded during concurrent electrode glomerular layer (GL) stimulations and light activation of the PmCo afferents reaching the GC layer [“EO” (E, electrical stimulation, + O, optical stimulation)]. Optogenetic stimulations were not delivered at frequencies higher than 20 Hz, to avoid ChR2 desensitization (Lin, 2011; Boyd et al., 2012). Each protocol was run for 5 min during which seal resistance was monitored. Typically after seal formation, mitral cells were left to stabilize for a few minutes before the recording started. Given the different duration of a single LED and electrical pulses (0.4 and 4 ms, respectively), the two stimuli were not overlapped. However, since the effect of LED stimulation on MC firing was evident on a wider scale (even seconds; Fig. 2*C*), we placed each 20 Hz LED train between the electrode 100 Hz train (40 ms duration) and the end of the following intertrain interval (∼200 ms), to cover the period in which both direct and indirect (rebound activity) MC responses have been previously observed (electrode train onsets: 81.2, 331.2, 581.2, 831.2 ms; wave form: offset from digitizer output = 0.5 ms, pulse duration = 0.4 ms, after pulse duration = 9.1 ms, total pulse duration = 10 ms; LED train onsets: 91.2, 141.2, 191.2, 241.2, 291.2 ms; wave form: offset from digitizer output = 10 ms, pulse duration = 4 ms, after pulse duration = 36 ms, total pulse duration = 50 ms). The firing rates (FRs) resulting from dual stimulations (EO) were compared with those evoked by LED stimuli alone (O) and calculated as previously described [EO = FRelectrode + LED − FRbaseline)/(FRelectrode + LED + FRbaseline), O = FRLED − FRbaseline)/(FRLED + FRbaseline); Boyd et al., 2012]. The relative effect of optogenetic stimulation of PmCo afferents (EO) on vomeronasal nerve (VN)-evoked responses (E) was calculated referring EO to VN-evoked frequency changes (E = FRelectrode − FRbaseline)/(FRelectrode + FRbaseline). Brains in which viral expression was found to be widespread outside the PmCo [in the MeA and bed nucleus of the accessory olfactory tract (BAOT)] were discarded and not included in this analysis.

### Statistics and data analysis

All ANOVAs are performed with SPSS software. The Bonferroni correction method was used for the *post hoc* tests, when applicable. All indicated data are expressed as the mean ± SEM.

**Table:** Statistical table

Experiment	Fig.	Test	Ind. var.	Factors	*F* value	Effect	*p* value
PRV tracing	Fig. 2A2	*t* test	PRV + cells	N.A.	N.A.	Genotype	0.018
MC (LED^−^)	Fig. 6C1	*p* value, *t* test	AP freq.	N.A.	N.A.	Stim.	0.019
MC (LED^+^)	Fig. 6C1	*p* value, *t* test	AP freq.	N.A.	N.A.	Stim.	0.002
Evoked resp.	Fig. 7B	*t* test	Amplitude	N.A.	N.A.	Cell type	0.0001
(f.stim vs f.post)	Fig. 8B	*p* value, *t* test	AP freq.	N.A.	N.A.	Stim.	0.05
MC (electrode)	Fig. 8B	Two-way ANOVA	AP prob.	*I*, R	*F*_(5,58)_ = 26.3	*I*	0.004
MC (electrode)	Fig. 8B	Two-way ANOVA	AP prob.	*I*, R	*F*_(1,58)_ = 2.6	R	0.112
MC (electrode)	Fig. 8B	Two-way ANOVA	AP prob.	*I*, R	*F*_(5,58)_ = 0.76	Interaction	0.622
Dual stim., type I MCs	Fig. 8F	Two-way ANOVA	AP rate	Protocol, cell type	*F*_(4,36)_ = 2.7	Interaction	0.04
EO/E plot	Fig. 8G	χ^2^	Distrib.	N.A.	N.A.	N.A.	2.4 × 10^−16^
EO/O plot	Fig. 8H	χ^2^	Distrib.	N.A.	N.A.	N.A.	3.5 × 10^−18^

f.stim, frequency during stimulation; f.post, post-stimulus frequency; resp., responses; distrib, distribution; *I*, current; R, resistance; freq, frequency; prob., probability; AP, action potential; N.A., not applicable.

## Results

### The posteromedial cortical amygdala sends corticofugal afferents to the AOB

The AOB is densely innervated by cortical amygdala output neurons (Raisman, 1972; Gutiérrez-Castellanos et al., 2014; Oh et al., 2014; Allen Brain Mouse Connectivity Atlas, experiment #114249084). However, their precise target localization and identity are unknown. To precisely identify the source of neuronal projections to the AOB, we first locally injected the retrograde tracer Ct-b (Fig. 1*A*). Precise targeting of the AOB, with very limited spread to the MOB (4 of 10 subjects; Fig. 1*B*), consistently resulted in dense labeling of layers II and III in the PmCo (Fig. 1*C*,*D*).

Layer-specific Ct-b injections revealed that Ct-b injections in the GC alone are sufficient to retrogradely label PmCo neurons (data not shown). However, since the tracer can be taken up by passing axon terminals also directed to MCs, this method is not valid to assess the specificity of PmCo targets. To better determine the layer specificity of amygdala corticobulbar projection (herein referred to as ACPs) afferents to the AOB, we next used a pseudotyped rabies virus as a conditional retrograde tracer (PRV; Wickersham et al., 2007; Fig. 1*E*). In this experiment, PRV was injected into the AOB of a mouse line in which the expression of the protein rabies-G [RABVgp4 (which is required for viral amplification and retrograde PRV trans-synaptic transport)] and the avian receptor tumor virus receptor A [TVA; (required for the virus to access the host cells)] were under *cre*-dependent control (*RABV* mice; Takatoh et al., 2013). *RABV* mice were crossed either with mice expressing *cre* recombinase under the control of the MC-specific promoter *Pcdh21* (MCs) or the *nNOS* (neuronal nitric oxide synthase) promoter, expressed by GC layer inhibitory neurons [GCs and main accessory cells (macs); Kosaka and Kosaka, 2007; Larriva-Sahd, 2008]. As neither *Pcdh21* nor *nNOS* are expressed in the PmCo (Fig. 1*F*), both TVA and G expression were limited to the injection site (Fig. 1*G1*,*H1*). This allowed only monosynaptic retrograde tracing (e.g., no PRV expression was found in areas two synapses away from AOB starter neurons, such as the hypothalamus or the hippocampus). Although it is possible that TVA/G can be expressed elsewhere due to *cre* expression outside the AOB [e.g., *Pcdh21* expression in the anterior piriform cortex (Nagai et al., 2005) and *nNOS* expression in the islands of Calleja, MeA, cerebellum, caudate putamen, cortex, hippocampus, hypothalamus], thus inducing neurons outside the AOB to be possible starters for PRV transport to the PmCo. However, this possibility could be ruled out as neither of these regions project to the AOB nor show PRV expression. Consistent with our Ct-b-tracing experiments, retrograde PRV labeling was found in several AOS regions, including the PmCo (Fig. 1*G2*,*H2*) and mainly from infection of *nNOS^cre+^* neurons (ratio of PRV^+^ cells PmCo/AOB: *Pcdh21^cre+^*, 0.04 ± 0.02; *nNOS^cre+^*, 0.54 ± 0.2; *N* = 4 brains for each strain, approximately five sections per animal, 1 section every 150 microns). These results confirmed that *nNOS*-expressing GC layer inhibitory neurons (GCs and macs), as opposed to MC neurons, are the major target of ACPs.

### Sublaminar specificity of PmCo–AOB reciprocal connections

Although, PRV-RABV allows for layer-specific retrograde tracing, through this approach is not possible to quantify the relative contribution of different neuronal types (GCs or macs) to retrograde PRV infection. This limitation also prevents the calculation of relative amounts of input neurons reaching these neurons from any brain area. To estimate the number of starter neurons in the AOB and the relative contribution of GCs and macs to the retrograde PRV infection, we used a complementary viral approach (Watabe-Uchida et al., 2012; Menegas et al., 2015) to conditionally express rabies-G and TVA-mCherry in *nNOS^cre^*-expressing neurons in the OB. This allowed for a more precise quantification of starter cells in the AOB, as those infected by PRV are GFP+ and those expressing the molecular component rabies-G and TVA are mCherry^+^ (Fig. 2*A*). Although double-labeled cells were found in both GCs and macs (Fig. 2*A1*), the majority were identified as GCs based on morphologic criteria (average percentage of total starters: GC 75,4%, macs 24,5%; *N* = 4; Fig. 2*A3*). Quantification of all PRV^+^ cells in the brains of infected animals showed consistent labeling in a restricted range of olfactory and limbic areas (Fig. 2*A4*). For each brain region, the relative percentage of traced neurons was calculated over the number of PRV cells collectively sampled in all brain areas (PRV-region/PRV-brain × 100; *N* = 4 brains, approximately four to five sections per animal, 1 tissue section every sixth animal). The amygdala alone gives rise to 9.7% of input neurons to AOB cre-expressing cells (Fig. 2*A4*). Of these, 83.5% are localized in the PmCo, with the majority arising from layer III (Fig. 2*A4*). These findings were consistent with our above Ct-b-tracing experiments (Fig. 1*C*,*D*). Overall, these results reveal that ACPs represent a major source of top-down feedback mainly targeting GCs in the AOB.

### Molecular phenotype and connectivity of ACPs

**Figure 3.**
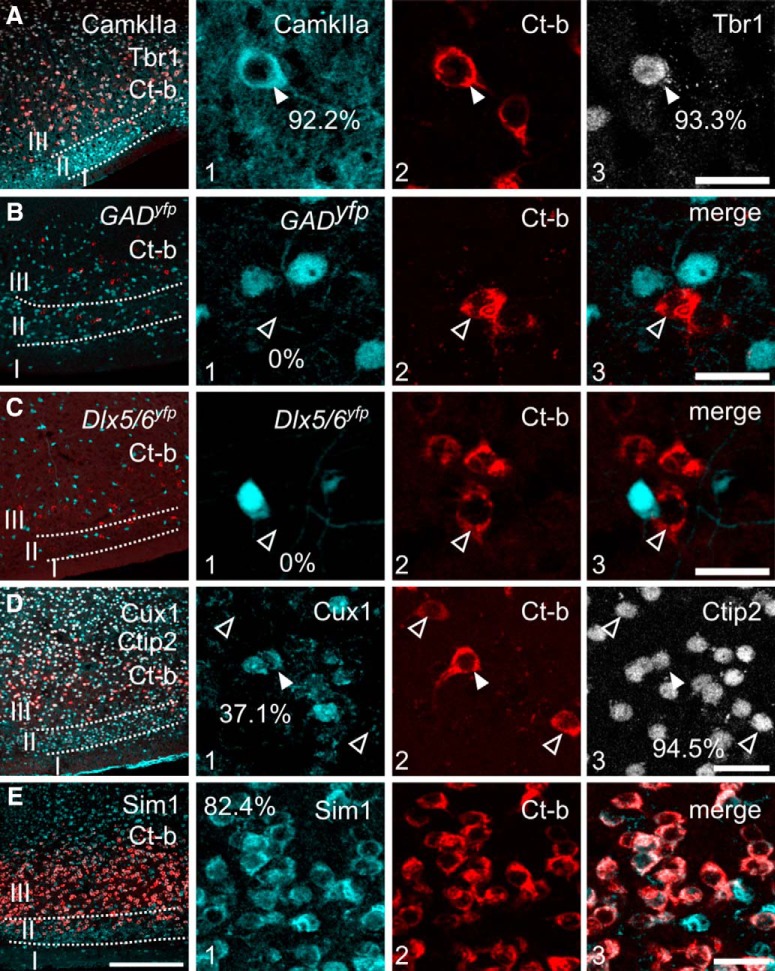
Molecular phenotypes of PmCo–AOB projection neurons. ***A***, PmCo corticobulbar projection neurons (ACPs) labeled with Ct-b after AOB retrograde tracing. Ct-b-labeled PmCo neurons (red) express the excitatory markers CaMKIIa (cyan, 92.2%) and Tbr1 (gray, 93.3%). ***B***, ***C***, Ct-b-labeled neurons in the PmCo do not express YFP in *GAD^cre^;Ryfp* or *Dlx5/6^cre^;Ryfp* mouse lines (cyan), confirming their excitatory phenotype. ***D***, PmCo-Ct-b-labeled neurons express Cux1 (cyan, 37.1%) and Ctip2 (gray, 94.5%), typical cortical neuron markers in layer II–IV and V and VI, respectively. All Cux1^+^ PmCo neurons coexpressed Ctip2. ***E***, Expression of the gene Sim1 reaches ca. 82.4% in PmCo Ct-b-positive cells. For each count, tissue collected (three to four sections) from three Ct-b-injected mice was used. Scale bars: left, 20 μm; right, 200 μm.

To define the molecular phenotype of ACPs, we conducted immunohistochemistry on tissue sections from AOB Ct-b-injected brains. We found that almost all PmCo Ct-b^+^ neurons coexpressed the excitatory neuronal markers CaMKIIa and Tbr1 (∼90%; Fig. 3*A*), with no coexpression of markers of inhibitory neurons such as GAD or Dlx5/6 (Fig. 3*B*,*C*). Retrogradely traced ACPs also expressed Ctip2 and Cux1 (∼30% overlap; Fig. 3*D*), similar to other subpopulations of corticobulbar neurons in the piriform cortex (Diodato et al., 2016). A large majority (82.4%) of Ct-b^+^ ACPs also coexpressed Sim1, a limbic system marker (Semple and Hill, 2018; Fig. 3*E*).

**Figure 4.**
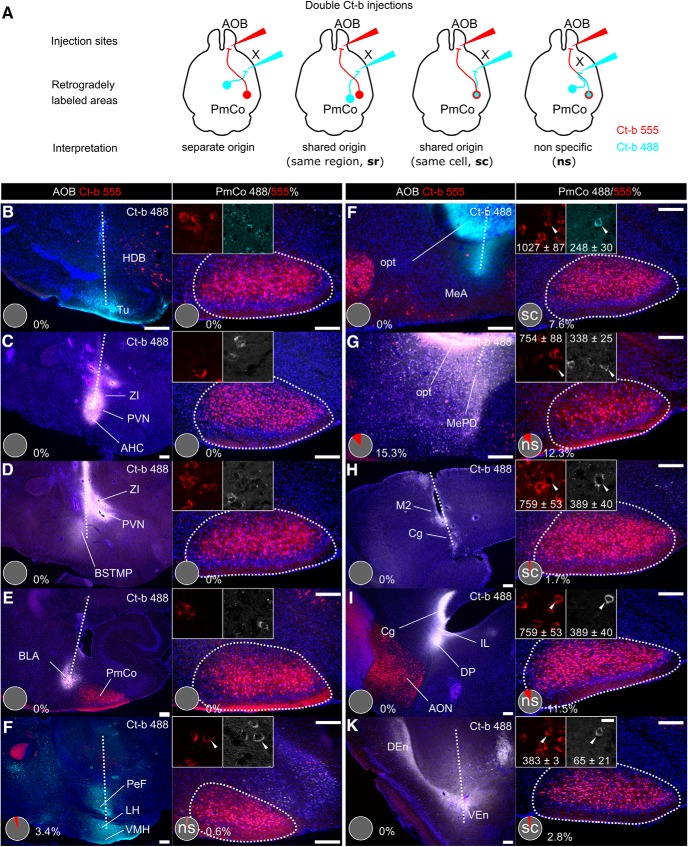
Collateral projections of PmCo corticobulbar neurons. ***A***, Dual Ct-b injections were used to identify possible additional target areas of the PmCo neurons retrogradely labeled from the AOB (Ct-b 555). Tracing was considered reliable in case of clear separation of the two injection sites and 555/488 colabeling of the same region (sr) or the same cells (sc). ***F–I***, We considered nonspecific (ns) tracing experiments to be those in which the two tracers showed partial overlap near the two injection sites or in case of Ct-b 488 injections adjacent to the stria terminalis (for reference, see Allen Brain Connectivity Atlas, experiment #114249084) where AOB-directed ACPs course (e.g., ***G***, ***I***): in such cases, Ct-b 488 would be likely taken up by passing fibers and yield false-positive results (compare ***H*** to ***I*** and ***G*** to ***F*** to see how Ct-b overlap in the PmCo decreases as the injection site is moved either dorsally or rostrally, respectively). ***B–H***, Injections of green Ct-b 488 (cyan) were targeted to different AOS regions, known main targets of the PmCo: the olfactory tubercle (Tu; ***B***), the paraventricular nucleus (PVN; ***C***, ***D***), the bed nucleus of the stria terminalis (BNST; ***D***), the ventromedial hypothalamic nucleus (VMH; ***E***), the MeA (***G***; MePD, ***H***), the basolateral amygdaloid nucleus (BLA, ***F***), the endopiriform nuclei (***K***), and the medial prefrontal cortex were targeted (***I***, ***J***). The pie charts in the panels showing the injections sites (left) indicate the coexpression of Ct-b 555 in Ct-b 488 fibers and therefore possible biases due to nonspecific tracing (tracing reliability is indicated according to the diagrams in A). Similarly, the coexpression of Ct-b 488 and Ct-b 555 in the PmCo is indicated in percentage in the panels on the right. In the regions showing higher coexpression, the absolute (averaged) values are indicated in the high-magnification insets. HDB, Nucleus of the diagonal band of Broca; ZI, zona incerta; BSTMP, bed nucleus stria terminalis medial division posterior part; PeF, perifornical nucleus; LH, lateral hypothalamic nucleus; opt, optic tract; M2, secondary motor cortex; Cg, cingulate cortex; IL, infralimbic cortex; DP, dorsal peduncular cortex; Den, dorsal endopiriform nucleus; VEn, ventral endopiriform nucleus. Scale bars: left panels, 20 μm; right panels, 200 μm. Data are the mean ± SEM.

Interestingly, corticobulbar projection neurons in the piriform cortex have been shown to extend axon collaterals to other subcortical and cortical targets (Diodato et al., 2016). This implies the existence of top-down inputs from other high-order olfactory areas such as the PmCo. Specific gene expression patterns in piriform corticobulbar projections have been associated with this top-down cortical circuit (Diodato et al., 2016). In particular, Cux1/Ctip2-expressing piriform cortex neurons have been shown to project to both the OB and areas of the prefrontal cortex (PFC; Diodato et al., 2016). Thus, to evaluate the presence of ACP axon collaterals to other brain regions, we injected Ct-b coupled with different fluorophores into both the AOB (Ct-b 555) and other known targets of PmCo efferent projections (Ct-b 488; Gutiérrez-Castellanos et al., 2014; Fig. 4*A*). We found unbiased 555/488 dual labeling only when the MeA (7.6%), the medial prefrontal cortex (mPFC; 1.7%), and the entorhinal cortex (Ent; 2.8%) were targeted together with the AOB (*N* = 3; Fig. 4*G*,*I*,*J*). Since no detectable differences were found using either tracer in single-Ct-b injection experiments, we are confident that ACPs mainly target the AOB with very limited collateral axonal projections to the MeA, Ent, and mPFC. This result also reveals similarities between ACPs and the subpopulations of other corticobulbar projection neurons in the piriform cortex (Diodato et al., 2016).

**Figure 5.**
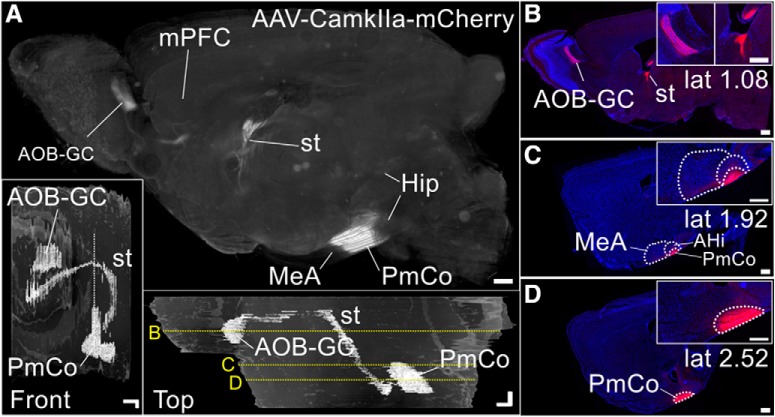
Anterograde tracing of PmCo projections using a CamKIIa-specific adenovirus. ***A***, Z-projection of an image stack acquired from seriate tissue sections and showing the extent of viral labeling in the brain (mCherry) following AAV injection in the PmCo: the corticobulbar tract coursing through the stria terminalis (st) and reaching the AOB GC layer shows the most intense mCherry expression. In the lower panels: digitized version of the image stack showing from frontal and top views the course of PmCo fibers in the brain. The pattern of mCherry expression was reconstructed on each image by selecting pixels having values of hue/intensity/brightness equal or higher than those in the AOB GC layer. In the frontal view, the route of the injection is indicated by the white dotted line. In the top view, the sectioning planes relative to the images in ***B***, ***C***, and ***D*** are in indicated by the dashed yellow lines. ***B–D***, Single images taken from the stack represented in ***A*** showing the extent of mCherry expression at the lateral levels indicated by the coordinates in the lower left corners of each panel. The AOB GC, the st, and the PmCo show the highest level of mCherry expression. Scale bars, 500 μm.

To further validate these findings, we performed anterograde viral-tracing experiments using a *CaMKIIa-*specific adeno-associated virus (AAV), exploiting the high expression levels of *CaMKIIa* in ACPs. When viral injections were restricted to the PmCo (*N* = 6; Fig. 5*A–D*), there was negligible or no viral expression in any targets of PmCO efferent projections such as the BAOT, basolateral amygdala, olfactory tubercle, and mPFC (Fig. 5*B–D*). Negligible or no evidence of viral expression was detected in the MeA, Ent, and PFC (all receiving minimal ACP collateral input; Fig. 4*F–K*), with the strongest expression in the AOB GC and along the stria terminalis (Fig. 5*A*,*B*). Overall, as shown by different retrograde and anterograde tracing methods, these results confirm that the AOB is the major target of ACPs.

### ACPs synapse onto AOB GABAergic interneurons

**Figure 6.**
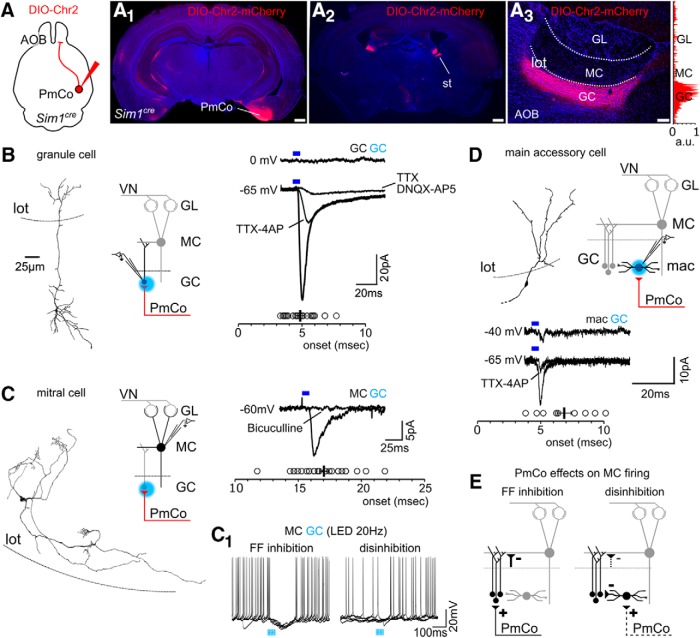
***A***, ACPs establish direct synaptic contact with AOB GCs. ***A_1_***–***A_3_***, Injection of double-floxed ChR2 mCherry-expressing adeno-associated virus in the PmCo (***A_1_***) of *Sim1^cre^* mice results in labeling (red) of the corticobulbar circuit coursing through the stria terminalis (st; ***A_2_***), and terminating in the AOB GC layer only (as shown by the color histogram on the side, ***A_3_***). ***B***, Light stimulation of PmCo afferents to the GC layer induced TTX-sensitive excitatory responses in GCs at resting potential (−65 mV; onset, 4.8 ± 0.2 ms; *N* = 23). These are rescued by TTX-4AP bath application, indicating direct synaptic connectivity. ***C***, No evoked post-synaptic current (EPSCs) is detected in MCs under the same conditions, while inhibitory currents (IPSCs; onset, 17.1 ± 0.3 ms; *N* = 28) are visualized using a high-chloride intracellular solution. ***C_1_***, Effects of repeated optogenetic stimulation of the GC layer on MC firing (five overlapped trials are shown for each effect): MC activity is either temporarily suppressed or facilitated (20 Hz light pulses, 4 ms each; frequency was compared 1 s before vs 1 s after LED stimulus onset). ***D***, The macs receive direct PmCo inputs of lower amplitude compared to GCs. ***E***, Circuit diagrams showing the putative effects of PmCo feedback on AOB MC firing: GC-mediated feedforward inhibition and mac–GC-mediated MC disinhibition. Above each trace, the recorded cell type and the site of LED stimulations are indicated in black and blue, respectively. a.u., Arbitrary units. Scale bars: ***A_3_***, 100 μm; ***A_1_***, ***A_2_***, 500 μm.

From our viral-tracing experiments, AOB GCs appear to be the main target of ACPs in the AOB. However, from this analysis it was not possible to evaluate the relative weight of ACP synaptic inputs onto either cell type or to assess the impact on the physiology of AOB circuits. To analyze these properties, we expressed ChR2 specifically in ACPs through conditional viral delivery in the PmCo of *Sim1^cre^* mice (*N* = 28; Fig. 6*A*), as Sim1 is expressed by the majority of these neurons (Fig. 3*E*). Four to six weeks after viral injection, ChR2 was strongly expressed in the PmCo (Fig. 6*A1*), along the stria terminalis (Fig. 6*A2*) and in the AOB GC layer (Fig. 6*A3*). Perisomatic stimulation with blue light evoked excitatory responses in GCs with relatively fast kinetics and low onset variability (4.8 ± 0.2 ms, *N* = 23), which is consistent with a direct excitatory input from the PmCo (Fig. 6*B*). This was further confirmed by 4-AP-mediated rescue of evoked excitatory events, initially blocked with TTX (onset, 8.3 ± 0.7 ms; amplitude reduction, 77.9 ± 23.2 pA; *N* = 6; Fig. 6*B*; Petreanu et al., 2009). Excitatory input was instead completely eliminated by blockers of AMPA and NMDA glutamatergic transmission, DNQX and AP5, respectively (Fig. 6*B*). The absence of light-evoked IPSCs recorded at the reversal potential for excitation (0 mV) indicated a lack of indirect inhibitory transmission between the PmCo and AOB GCs (*N* = 23; Fig. 6*B*). MC layer or GL light stimulation did not result in any response (neither excitatory nor inhibitory) in either GCs (*N* = 23) or MCs (*N* = 28). Conversely, light activation of PmCo afferents to the GC layer evoked disynaptic IPSCs in MCs (Fig. 6*C*). These responses were approximately three times slower than those evoked in GCs (onset, 17.1 ± 0.3 ms; *N* = 28) and completely abolished by bicuculline, revealing their polysynaptic nature, likely resulting from GABA release from PmCo-activated GCs. Accordingly, trains of light pulses on GCs (4 ms at 20 Hz) induced distinct effects on MC firing (holding = −45 mV): either a sharp and transient decrease (compare 70% reduction: 1 s before LED vs 1 s after LED; found in *N* = 9 of 27 cells; paired *t* test before vs after, *p* = 0.019) or a gradual increase in the normalized spike frequency (∼30% increase: 1 s before LED vs 1 s after LED, found in *N* = 4/27 cells; paired *t* test before vs after, *p* = 0.002; Fig. 6*C1*).

In the MOB, deep short axon cells (dSACs) are a type of inhibitory GABAergic neuron that provides feedforward inhibition to multiple GCs. dSACs are also the main recipient of Pir excitatory feedback, which in turn results in strong GC inhibition (Boyd et al., 2012; Markopoulos et al., 2012). Therefore, while direct GC-mediated inhibition can result in a reduction of MC firing rate, both dSAC-mediated disinhibition and GC-induced rebound firing (Desmaisons et al., 1999; Balu and Strowbridge, 2007) can explain the slow increase in MC firing we observed. In the AOB, macs have an analogous connectivity and function as dSACs (Larriva-Sahd, 2008). Surprisingly, the activation of PmCo afferents on macs (onset, 6.9 ± 0.6 ms; *N* = 10 cells) elicited excitatory events much lower in amplitude when compared to GCs (amplitude: GCs, 103.1 ± 21.9 pA, *N* = 23; macs, 20.9 ± 3.1, *N* = 10; *t* test, *p* = 0.005; Fig. 6*B*). The persistence of very low-magnitude responses detected in the presence of TTX and 4-AP (Fig. 6*D*) revealed the occurrence of direct synaptic connectivity with PmCo afferents; however, under these conditions (perisomatic or wide-field LED illumination), they were not sufficient to induce detectable light-evoked feedforward inhibition of GCs (Fig. 6*B*).

**Figure 7.**
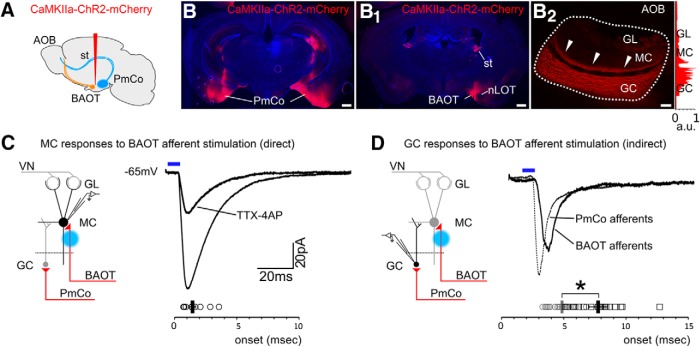
Optogenetically evoked MC excitatory responses are induced by the activation of BAOT afferents. ***A–B_2_***, Spread of *CaMKIIa-ChR2* virus to the BAOT results in the expression of ChR2 not only in the PmCo projection neurons but also in those in the BAOT. Infection of this region results in mCherry labeling in the AOB GC and MC layers (see color histogram on the side, indicating the localization of mCherry labeling). ***C***, Optogenetic stimulation of the MC layer evoked excitatory responses in MCs (onset, 1.3 ± 0.2 ms; 14 cells) only when the BAOT was infected. ***D***, These direct responses (not blocked by TTX-4AP bath application) result in disynaptic excitatory events detectable in GCs, which are slower (onset, 7.7 ± 0.4 ms; 17 cells) compared with PmCo direct inputs elicited by GC stimulation (4.8 ± 0.2 ms; 23 cells; *t* test, *p* < 0.0005; Fig. 1*B*).

Given that our tracing experiments suggested a lower extent of PmCo–MC connectivity (Fig. 1*G2*), the lack of light-evoked EPSCs in MCs was unexpected. One possibility is that PmCo projections to MCs are either *Sim1* ^−^ or simply too scarce to be detected. To rule out these possibilities, we used a *CaMKIIa-*specific viral vector as described above to target ChR2 expression to the highest possible number of corticobulbar projection neurons in the PmCo (CaMKIIa/Ct-b = 92.2%; Fig. 3*A*). In this case, during perisomatic LED stimulations, direct and fast excitatory responses were sometimes detectable in MCs (onset, 1.3 ± 0.2 ms; Fig. 7). However, by a thorough survey of all injected brains used in these experiments, we were able to rule out the origin of excitatory afferents to MCs in the PmCo: fast monosynaptic excitatory currents were detected only when *CaMKIIa*-expressing neurons in the BAOT—which also projects to the AOB (Fig. 1*D*)—were also infected (Fig. 7; Table 1). No other types of excitatory events (slower in onset) were detected on MCs. Collectively, these results conclusively validate the observation that ACPs innervate the AOB GC layer only and further confirm that this input is mainly directed to AOB GCs (Fig. 6*E*).

**Table 1:** Summary of CaMKIIa optogenetic stimulations experiments

AAV expression		Evoked EPCs		
PmCo	BAOT	MC	GC	mac
Yes	N/D	No	Yes	No
No	N/D	Yes	N/D	
No	N/D	Yes	No	
Yes	N/D	Yes	No	
Yes	N/D	Yes	Yes	Yes
Yes	No	N/D	Yes	
Yes	No	N/D	Yes	
Yes	No	N/D	No	
Yes	No	N/D	N/D	Yes
Yes	No	No	Yes	
Yes	No	No	Yes	
Yes	No	No	N/D	
Yes	No	No	No	
No	No	No	No	
Yes	No	No	N/D	
No	No	No	No	
Yes	No	No	Yes	
Yes	No	No	Yes	
Yes	No	No	N/D	Yes
Yes	No	No	Yes	
Yes	Yes	N/D	N/D	
Yes	Yes	Yes	No	
Yes	Yes	Yes	N/D	
Yes	Yes	Yes	N/D	
Yes	Yes	Yes	Yes	
Yes	Yes	Yes	N/D	Yes
Yes	Yes	Yes	Yes	Yes
Yes	Yes	Yes	N/D	Yes

Viral delivery of Chr2 to the PmCO was accomplished using a CaMKIIa-specific viral vector (see Materials and Methods) to target excitatory neurons. When the PmCo was efficiently targeted (first left column, PmCo, value = yes), excitatory responses were evoked in AOB GCs. Similar responses were observed also in AOB MCs (MC) but only when the BAOT was labeled (second left column, BAOT, value = yes; *N* = 7). In other cases (*N* = 4) MCs were also responsive to perisomatic light stimulations but, due to tissue damage during brain tissue harvest, the area corresponding to the anterior portion of the injection site (in proximity with the BAOT) was not available. This survey was used to assess the likelihood of PmCo-to-GC and BAOT-to-MC connectivity.

### ACPs enhances AOB mitral cell excitatory output

**Figure 8.**
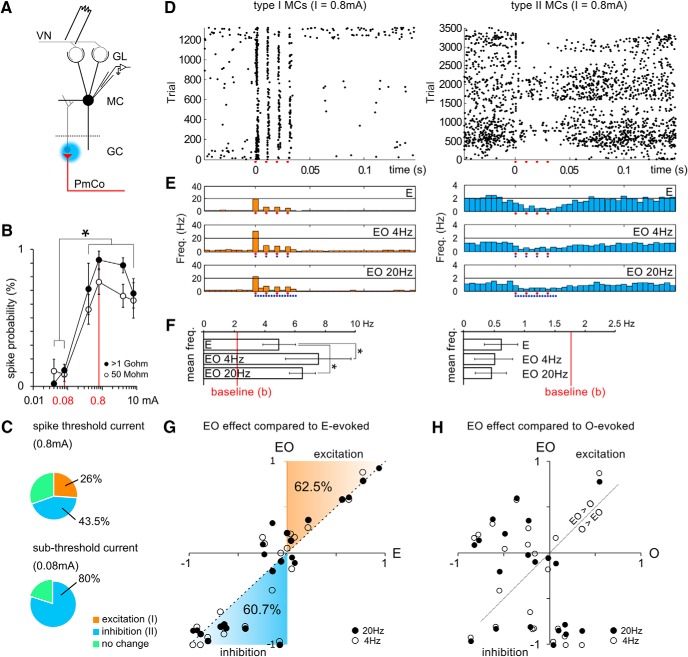
PmCo feedback enhances MC responses to vomeronasal inputs. ***A***, Diagram showing the configuration used during recordings: electrical stimuli were targeted onto the VN, light stimuli were delivered onto the GC layer, and the firing rate of MCs was recorded in cell-attached mode. ***B***, Tuning curves of MCs in response to current stimuli of different intensity were obtained at different seal resistance levels: 1 GΩ, black dots; 50 MΩ, circles. This preliminary test was made to define the threshold current for MC firing, used in the rest of the experiments (2-way ANOVA, *p* = 0.004). ***C***, Percentage of the different types of MC responses following electrical VN stimulations above (0.8 mA) and below (0.08 mA) threshold. ***D***, Raster plots of type I and type II MC activity during electrode stimulation (red dots). ***E***, Average firing rate (across units and trials) of MCs divided by type (I or II) and stimulation. EO 4 Hz/EO 20 Hz (joint electrical and optical stimulation at 4 and 20 Hz, respectively. Red dots mark electrical stimuli; blue dots mark optical stimuli. ***F***, Mean firing rate changes induced in MCs by optogenetic activation of PmCo feedback. The red lines represent the firing frequency at unstimulated baseline levels. ***G***, Relative increase and decrease in firing rates during joint electrical and optical stimulation (*y*-axis) and electrical only stimulation (*x*-axis) both referred to baseline levels. Firing rate was computed during 40 ms of stimulation. ***H***, Two LED stimulation protocols are color coded in darker (EO 20 Hz) or lighter (EO 4 Hz) red (for excitation) or blue (for inhibition). Percentage values refer to either values of E > 0 or E < 0). Scatter plot showing the lack of correlation between the relative effect of dual electrical and light stimulations on light-evoked responses alone. Data are the mean ± SEM.

In the MOB, GC-mediated inhibition has been proposed to be responsible for tuning MC responses to different odor inputs by sharpening their molecular receptive range through the suppression of nonspecific neuronal responses and facilitating relevant output (i.e., providing contrast enhancement; Yokoi et al., 1995; but, see also Fukunaga et al., 2014). *In vivo* experiments have shown that AOB MC firing can either increase or decrease in response to different odor stimuli (Luo et al., 2003), suggesting the presence of similar tuning mechanisms also in the AOB. In our experiments, corticofugal PmCo inputs induced either inhibitory or disinhibitory effects on AOB MCs (Fig. 6*C1*), which may potentially indicate a contribution to MC odor coding through contrast enhancement. To study this further, we conducted cell-attached recordings from AOB MCs during concurrent electrical stimulations of the VN and blue light excitation of PmCo afferents (Fig. 8*A*). VN stimulations consisted of a series of 4 × 100 Hz trains of 0.4 ms pulses delivered in 2 s trials (Schoppa, 2006). Light stimuli were partially interleaved with electrical pulses and consisted of 4 × 4/20 Hz trains of 4 ms light pulses delivered onto PmCo afferents in the AOB GC layer. Most cells (16 of 23 cells) were responsive to a single electrical pulse, as the current used for the stimulations was tuned each time to reach firing threshold [≥0.4 mA for both low (50 MΩ) and high (1 GΩ) resistance seals; two-way ANOVA, factors: current (levels: 0.04, 0.08, 0.4, 0.8, 4, and 8 pA); resistance (levels: 1 GΩ, 50 MΩ); current effect, *p* < 0.005; significant pairwise *post hoc* comparisons (*p* < 0.05): 0.04 and 0.08 pA vs 0.4, 0.8, 4, and 8 pA; Fig. 8*B*]. As expected, AOB MCs showed either excitatory or inhibitory responses to VN input stimulation alone (Fig. 8*C*,*D*). Comparing the firing rates during and after VN stimulations (40 ms ON vs 200 ms OFF), we selected excited (type I) and inhibited (type II) cells to further analyze the effect of PmCo feedback in relation to different VN inputs (paired *t* test, *p* ≤ 0.05; Fig. 8*D*). This categorization accounted for the relative changes in firing rates between baseline activity and evoked responses and was referred to a more restricted sample of cells (16 of 23 cells). Inhibited cells were the most represented [type II cells were ∼50% (10 of 23 cells) while type I cells only were ∼26% (6 of 23 cells)], probably due to the high number of inhibitory neurons recruited by electrode stimulations of the VN. Cells that had responses that did not fall into either of the two categories were considered not to have any statistically significant change in firing rate (“no change”; Fig. 8*C*). When current intensity was kept at subthreshold levels (0.08 mA), only type II MCs were observed (Fig. 8*C*). Together, these results suggest that the threshold for MC excitability is determined by both VN input and the extent of concurrent activation of local inhibitory circuits.

When trains of light stimuli were delivered to PmCo afferents in the GC layer, the type I MC firing rate was increased on concurrent VN afferent stimulation, while type II MC responses remained low in frequency or were even slightly reduced (∼2 Hz; two-way ANOVA, factors: type (levels: I, II, no change); protocol (levels: E, EO 4 Hz, EO 20 Hz); interaction effect: *F*_(4,36)_ = 2.78, *p* = 0.04; type effect: *p* < 0.005; protocol effect: *p* = 0.03; *post hoc* comparisons: E vs EO 4 Hz, *p* = 0.014; E vs EO_2_, 0 Hz, *p* = 0.018; type I, *N* = 4; type II, *N* = 10, no change, *N* = 7; Fig. 8*E*,*F*). In other words, considering only the two subsets of MCs characterized by significant VN-evoked firing rate changes (type I, II), the addition of optogenetically evoked PmCo input was mainly evident in the type I MCs. This implies that the predominant inhibitory effect of PmCo feedback on MCs observed during voltage-clamp experiments (Fig. 6*C*,*C1* ) might be limited by the excitatory effects induced by the concurrent activation of VN afferents in both type I and II responses. Conversely, since both the activation of PmCo corticofugal afferents as well as electrical VN stimulation can induce GC-mediated rebound excitation (Schoppa, 2006), or other disinhibitory mechanisms enacted by local GABAergic circuits, these two effects might be additive in other cases, possibly explaining the more significant effect of dual stimulations on type I responses. Thus, in contrast to the generic and homogeneous impact of piriform afferents to MOB circuits (Boyd et al., 2012; but, see also Otazu et al., 2015), the effect of PmCo feedback depends on the polarity of VN-evoked responses in AOB MCs. Accordingly, photoactivation of PmCo afferents did not shift MC firing rates toward excitation during subthreshold VN stimulations (data not shown). However, this analysis was limited to the two extremes of the VN response range (type I and type II). To test whether the effect of dual PmCo/VN activation could be generalized to all VN-evoked responses, we compared the relative frequency changes [(F_evoked_ − F_baseline_)/(F_evoked_ + F_baseline_); see Materials and Methods] during VN stimulations (electrode-evoked vs baseline firing rates = E) to those evoked during dual VN/PmCo stimulations, in all recorded MCs (electrical + optical stimulation = EO). Firing rates during dual stimulations were either shifted toward excitation (in 62.5% of all cells for which F_EO_ > F_baseline_, F_EO_ > F_E_) in case of positive VN-evoked responses or inhibition (in 60.7% of all cells for which F_EO <_ F_baseline_, F_EO_ < F_E_) in case of negative ones (E < 0; χ^2^ tests were run to compare the effect of stimulations to random data distributions yielded *p* values <0.0001). Thus, the effect of PmCo input on MC gain to VN-evoked activity appears to be conserved in most recorded neurons in our sample (Fig. 8*G*). Importantly, the differential effect of cortical input on MC VN-evoked firing does not depend on PmCo input alone since very low correlation was found (*R* ^2^ = 0.2) comparing the effect of dual stimulations (EO) to the one of light stimulations alone (O; Fig. 8*H*). Light-evoked firing rate changes (in absence of paired electrical stimulations) were broadly inhibitory (F_O_ < F_baseline_ = 65.8%; similar rates were found in current-clamp experiments: N_inhibited_/(N_inhibited_ + N_excited_) × 100 = 69.2%). Together, these results indicate that PmCo feedback exerts differential and input-specific effects on MCs. This leads to an increase in the gain of MC responses to incoming stimuli, which is a typical functional requirement for odor discrimination by olfactory circuits. These data, together with our electrophysiological analysis, suggest that, similar to what occurs in piriform circuits, amygdala corticobulbar neurons might play a crucial role in shaping odor processing by the AOS through experience or brain state-dependent feedback.

## Discussion

In this study, we dissected the functional connectivity of a corticobulbar circuit originating in the PmCo and innervating the AOB. We show that the PmCo receives direct input from the AOB and in turn establishes direct synaptic connections with AOB GABAergic neurons, eliciting feedforward modulation of MC firing. Optogenetic activation of PmCo corticofugal afferents during stimulation of VNO input to the AOB enhances MC output activity, indicating a possible role of amygdala corticofugal circuits in odor processing by the AOS.

### Functional dissection of the PmCo corticobulbar circuit

From our Ct-b- and retroviral-tracing experiments, we find that higher-order brain input to the AOB mainly originates in the PmCo. At the synaptic level, our experiments show that optogenetic stimulation of the PmCo–AOB afferents evokes direct excitation onto GC layer neurons only. This finding is consistent with previous studies showing that the predominance of piriform cortical and amygdala centrifugal inputs is directed to the granule cell layer (Balu et al., 2007; Matsutani, 2010; Gutiérrez-Castellanos et al., 2014). Although PRV infections in the AOB of *Pcdh21^cre^* mice yielded some retrograde tracing to the PmCo, suggesting connectivity between PmCo and MCs, we believe that this might be due to either recombination leakiness in the *RABV* mouse or nonspecific PRV transport (the occurrence of which was minimal even in the absence of Cre expression and could be caused by local leakage of helper adeno-associated viruses; Miyamichi et al., 2013) rather than by direct connectivity. The fact that neither *Sim1^cre^*- nor *CaMKIIa*-driven conditional ChR2 expression limited to the PmCo led to MC activation supports this interpretation.

### Role of PmCo feedback on AOB circuit activity

Compared with the PmCo–AOB circuit analyzed here, corticobulbar projections from the piriform cortex to the MOB display an analogous connectivity. Within the granule cell layer, piriform afferents mainly reach MOB dSACs (Boyd et al., 2012). These have been proposed to regulate MC inputs in a center-surround fashion acting via ensembles of connected interneurons (Willhite et al., 2006; Kim et al., 2011; Geramita et al., 2016). GCs instead provide a more narrowly tuned inhibitory drive onto MCs (Boyd et al., 2012). In the AOB, we find that the amount of excitation delivered to macs (homologous to dSACs; Larriva-Sahd, 2008) by PmCo afferents is much lower compared with the input onto GCs (Fig. 6), and we never observed inhibitory responses in GCs on light activation of PmCo afferents, as would occur in the case of strong and diffuse PmCo–macs–GC connectivity. This could indicate a narrower tuning of corticofugal circuits directed to the AOB and the presence of a lower degree of lateral interactions between MCs and GCs (Castro et al., 2007; Moriya-Ito et al., 2009; but, see also Guo and Holy, 2007; Hendrickson et al., 2008). Consistent with this view, periglomerular cells—the very first layer of horizontal integration of incoming input to MCs—are scarcer in the AOB than in the rest of the bulb (Meisami and Bhatnagar, 1998). In addition, AOB GCs are mainly connected to MC apical dendrites (Castro et al., 2007; Moriya-Ito et al., 2009), where they likely shunt inputs converging on a single MC rather than regulating adjacent mitral cell circuits, as in the MOB (Geramita et al., 2016). It follows that GC-mediated selective inhibition—as opposed to rebound excitation or mac-mediated disinhibition—of MC output might be the dominant mechanism by which inhibitory feedback triggered by PmCo projections regulates MC activity in the AOB. This view is supported by the fact that MC responses to VN stimuli are shifted more toward suppression (type II) than excitation (type I; Fig. 8*C*): type II MC responses represent 43% of all MC VN-evoked responses, while type I represent only 26% (indicating a marked recruitment of local feedback inhibitory circuits in the response to VN stimuli). Because the effect of ACP feedback does not change the ratio of the response type (Fig. 8G), their effect on MC gain is also more tuned toward suppression, if one considers the whole sample of recorded cells (not only type I and II).

Therefore, our results are in agreement with a GC-centered wiring of PmCo projections (as opposed to the dSAC-centered organization of piriform afferents in the MOB). These inputs are likely to act preferentially on a much narrower scale, consistent with their hypothesized role in tuning the highly selective odor responses of AOB MCs. Conversely, in the MOB, the broader innervation of inhibitory circuits by piriform afferents might explain their more generalized inhibitory action on MC firing, with limited or no dependence on incoming peripheral stimuli (Boyd et al., 2012). In this case, given the broader tuning of MOB MCs to single-odor molecules, the coding fidelity of odor information might be achieved by coordinating the activity of larger ensembles of MCs, properly matching their activity patterns to different odor inputs (e.g., decorrelating odor responses; Otazu et al., 2015). Conversely, in the AOB this correspondence might be theoretically more precise since MCs receive a highly specific but heterogeneous set of inputs. However, due to their heterotypic glomerular connectivity, overlap might exist in the set of inputs that each MC is tuned to. We propose that the functional organization of the PmCo corticobulbar pathway is suitable to improve coding fidelity through contrast enhancement of odor representations by a very limited set of MCs.

### Role of amygdala corticofugal circuits in the encoding of social signals

Our results reveal in detail that the cortical amygdala and the AOB are directly interconnected. This has two important implications: first, the amygdala modulates the early processing of sensory information through corticobulbar input; and, second, this modulatory role might occur early (i.e., in the AOB) before any valence-related processing by either the amygdala or parallel circuits. The amygdala circuits such as the medial, basal, and central nuclei might be implicated in the further elaboration of value and the motivational aspects of these inputs (Moncho-Bogani et al., 2005; DiBenedictis et al., 2015; McCarthy et al., 2017).

Within the MOS, the most prominent cortical top-down modulation occurs through corticofugal projections from the piriform cortex, which provides a crucial feedback for the earliest stages of olfactory processing (Boyd et al., 2012; Otazu et al., 2015). The organizational similarities shared by these pathways and the PmCo corticobulbar circuits highlighted by our study, not only confirm the proposed role of the PmCo as the primary vomeronasal cortical area (Mucignat-Caretta et al., 2006; Gutiérrez-Castellanos et al., 2014), but also suggest that these inputs may be instrumental to the attribution of behavioral relevance to only a selected range of signals. This would imply a role of ACPs in the fine tuning of highly selective AOB responses to social odors. However, since some ACPs send axon collaterals to the MeA, PFC, and Ent (Fig. 5), these could play a more complex role than simply shaping odor processing by the AOB.

Importantly, a recent study showed that chemogenetic silencing of the MeA results in impairments in social odor processing (expressed as a decreased difference in male vs female odor investigation shown by female subjects; McCarthy et al., 2017), likely due to defects in receptive behaviors and in the motivation to investigate sex odors, rather than solely sensory deficits (DiBenedictis et al., 2015). Indeed, since MeA output mainly targets hypothalamic nuclei involved in mid- to long-term hormonal, motivational, and consummatory consequences of social odor perception (Bian et al., 2008; Bergan et al., 2014), it appears that ACP–AOB collaterals to the MeA might have more of a relay function rather than tuning incoming odor input.

In addition, although both the prefrontal cortex (Li et al., 2010) and the entorhinal cortex (Mayeaux and Johnston, 2004; Hargreaves et al., 2005) have been implicated in the encoding of key aspects of odor perception, lesion studies revealed that these areas might be dispensable for odor discrimination (Koger and Mair, 1994; Mayeaux and Johnston, 2004). Thus, both may be more involved in the multimodal elaboration of odor-associated inputs and odor value (Schoenbaum et al., 1999; Rolls, 2001; Alvarez and Eichenbaum, 2002; Chapuis et al., 2013; Ferry et al., 2015). The PmCo, given its direct connections to the AOB, can process relevant odor information within the same time scale of primary odor processing (Mucignat-Caretta et al., 2006; Maras and Petrulis, 2008), with ACPs mainly providing a direct feedback to the earlier steps of AOB-mediated sensory processing, as opposed to other value-associated functions. ACPs might also integrate more complex information, related to brain states or aspects of social odor perception. Because ACPs are reminiscent of piriform–MOB connections, our data contribute to underline the importance of direct and fast cortical input relaying brain state-related information back to all primary olfactory circuits to optimize odor perception (Boyd et al., 2012; Markopoulos et al., 2012; Rothermel et al., 2014; Otazu et al., 2015; Smith et al., 2015).

This poses the need of rethinking olfactory-based responses as functions that are integrated at a system level, with significant cross talk and feedback interactions, as opposed to being simply the outcome of unidirectional computations by segregated olfactory or amygdala subcircuits. Future studies are required to extend this concept to other sensory systems and to understand how the valence and saliency of social cues might develop or change, adapting to different brain states or pathophysiological conditions.

## Synthesis

Reviewing Editor: Margaret McCarthy, Univ of Maryland School of Medicine Decisions are customarily a result of the Reviewing Editor and the peer reviewers coming together and discussing their recommendations until a consensus is reached. When revisions are invited, a fact-based synthesis statement explaining their decision and outlining what is needed to prepare a revision will be listed below.

The authors have appropriately revised the manuscript in response to the review received from eLIFE. In particular, dropping the behavioral experiments and focusing only on the circuit analysis makes for a clear and strong report. There are however some typo's that if corrected would enhance readability. They are listed below, un-corrected. Unfortunately since there were no page numbers on the document they are not annotated but they are listed in the order they appear.

TYPOS

However, since the tracer can be uptaken by passing by axon terminals also

However, we can rule out this possibility as neither of these regions are projecting to the AOB or did show PRV-expression

Specific gene expression patterns in piriform corticobulbar projections have been associated this top-down cortical circuit

Cells which responses did not fall into either of the two categories were considered to not have any statistically significant change in firing rate

Chi-square tests were run to compare the effect of stimulations to random data distributions yielded p-values lower than 0.0001

These, together with our electrophysiological analysis, constitute an important evidence that

but also suggests these inputs maybe instrumental to the attribution of behavioral relevance to only a selected range of signals.

Importantly, a recent study showed that chemogenetic silencing of the MeA results impairments in social odor processing

it seems likely that ACP-AOB collaterals to the MeA might have more a relay function rather than tuning incoming odor input.

with significant cross-talk and feedback interactions, as opposed to be simply the outcome of unidirectional computations by segregated olfactory or amygdala sub-circuits

## Author Response

Dear eNeuro Reviewing Editor,

We are excited that our manuscript has been found potentially suitable for publication in eNeuro. Thank you for your careful review and noticing the typos and grammatical errors. We have now corrected those errors as well as other small errors in grammar that we noticed upon re-reading.

Sincerely,

Joshua Corbin Livio Oboti
